# Development of a core outcome set for traumatic brachial plexus injuries (COMBINE): study protocol

**DOI:** 10.1136/bmjopen-2019-030146

**Published:** 2019-06-14

**Authors:** Caroline Miller, Jane Cross, Dominic M Power, Derek Kyte, Christina Jerosch-Herold

**Affiliations:** 1 School of Health Sciences, University of East Anglia, Norwich, UK; 2 University Hospitals Birmingham NHS Foundation Trust, Physiotherapy Department, Queen Elizabeth Hospital, Birmingham, UK; 3 The Birmingham Peripheral Nerve Injury Service, Queen Elizabeth Hospital Birmingham, Birmingham, UK; 4 Centre for Patient-Reported Outcomes Research, Institute of Applied Health Research, University of Birmingham, Birmingham, UK; 5 NIHR Birmingham Biomedical Research Centre, University Hospitals Birmingham NHS Foundation Trust, Birmingham, UK

**Keywords:** brachial plexus, core outcome set, qualitative research, consensus development, outcome measures

## Abstract

**Introduction:**

Traumatic brachial plexus injury (TBPI) involves major trauma to the large nerves of the arm which control the movement and sensation. Fifty per cent of injuries result in complete paralysis of the arm with many other individuals having little movement, sensation loss and unremitting pain. The injury often causes severe and permanent disability affecting work and social life, with an estimated cost to the National Health Service and the economy of £35 million per annum. Advances in microsurgery have resulted in an increase in interventions aimed at reconstructing these injuries. However, data to guide evidence-based decisions is lacking. Different outcomes are used across studies to assess the effectiveness of treatments. This has impeded our ability to synthesise results to determine which treatments work best. Studies frequently report short-term clinical outcomes but rarely report longer term outcomes and those focused on quality of life. This project aims to produce a core outcome set (COS) for surgical and conservative management of TBPI. The TBPI COS will contain a minimum set of outcomes to be reported and measured in effectiveness studies and collected through routine clinical care.

**Methods and analysis:**

This mixed-methods project will be conducted in two phases. In phase 1 a long list of patient-reported and clinical outcomes will be identified through a systematic review. Interviews will then explore outcomes important to patients. In phase 2, the outcomes identified across the systematic review, and the interviews will be included in a three-round online Delphi exercise aiming to reach consensus on the COS. The Delphi process will include patient and healthcare participants. A consensus meeting will be held to achieve the final COS.

**Ethics and dissemination:**

The use of a COS in TBPI will increase the relevance of research and clinical care to all stakeholders, facilitate evidence synthesis and evidence-based decision making. The study has ethical approval.

**Trial registration numbers:**

CRD42018109843.

**Strengths and limitations of this study**

- A core outcome set will facilitate evidence synthesis in traumatic brachial plexus injury.
- A systematic review will identify the outcomes reported in recent trials and observational studies.
- Patient interviews will ensure that outcomes important to patients are identified.
- The Delphi and consensus meeting will include international patient and professional participants.
- Future research will be needed to agree a measurement instrument for each outcome in the core outcome set

## Introduction

A traumatic brachial plexus injury (TBPI) is an insult to the whole or part of the brachial plexus. A TBPI typically occurs in young men in their 20s and early 30s,^1–4^ and the majority result from motor vehicle accidents.^3–6^ Many patients have permanent denervation to the muscles in the arm, which results in complete paralysis, significant life-long pain and disability.^6–8^ TBPI accounts for 1.2% of polytrauma.^5 9^ Although the injury is relatively rare, its complex and chronic nature is associated with a combined National Health Service and ongoing economic cost of £35 million annually.^10^ Moreover, the incidence and costs are rising with improved survival following major trauma^11 12^ and associated increase in injury complexity^13^ resulting in significant societal and economic burden.

Recent advances in nerve microsurgery have increased the available reconstructive options.^14^ Studies evaluating outcomes from surgery focus on short-term clinician-reported outcomes such as early recovery of strength and movement.^2 15–19^ Yet final outcome following a TBPI or an associated intervention may not be seen until after 3 years. In addition, outcomes prioritised by patients, such as pain^8^ social disability^7 20 21^ and return to work^22 23^ are rarely measured,^19^ resulting in a mismatch between what outcomes patients and clinicians prioritise as important. Furthermore, studies of TBPI commonly collect and report differing outcomes which are often poorly defined, making it impossible to compare or combine results.^19^

The Clinical Advisory Group on services within major trauma recommend that more research is conducted on outcome measurement.^24^ Specifically, there is international agreement that a battery of outcome measures is necessary to assess all aspects of the impact of a TBPI on the individual.^19 25^ Moreover, the UK patient group (TBPI Society UK) has highlighted the need to focus on outcomes such as pain, emotion and psychology.^26^ Patient and public consultation, in preparation for this project, also revealed that patients felt that the broader impact of a TBPI on an individual was not addressed by current outcome assessment.

One solution is to develop a core outcome set (COS) for surgical and conservative management of TBPI. A COS contains a minimum agreed set of outcomes to be reported and measured in all studies and collected through routine clinical care.^27–29^ A number of COS in different clinical areas have been developed and are supported by Cochrane Collaboration reviewers and research funders.^30 31^ COS methodology is being refined and promoted by the Core Outcome Measures in Effectiveness Trials (COMET) initiative.^32^

A core outcome set for TBPI, derived through collaboration between patients and clinicians/researchers, will ensure that future studies collect outcomes that are meaningful to patients and address all key concerns.^33^ If outcomes are standardised, then interventions can be fully evaluated while minimising outcome-reporting bias.^34^ Using a COS to monitor clinical outcomes will facilitate consistent benchmarking of services and identify opportunities to raise clinical practice standards. A search of the COMET initiative database (http://www.comet-inititative.org/) was completed prior to commencing the project. No published or ongoing COS for clinical practice or effectiveness trials involving patients with traumatic brachial plexus injuries were identified.

### Aim

The aim of the project is to produce a COS for TBPI relevant to patients, healthcare professionals and academics.

### Scope

The scope of this COS is intended for international routine clinical care and clinical studies evaluating the effectiveness of interventions (surgical and conservative) for adults with a TBPI.

### Health condition

TBPI.

### Population

Adults >18 years of age.

### Types of interventions

All surgical and non-surgical interventions.

## Method

The development of this COS adheres to COMET and COS-Standards for Development (COS-STAD) recommendations.^35 36^ The study is a mixed-method design involving the use of both qualitative and quantitative methodologies. A Delphi study informed by data from the systematic review and qualitative interviews, and a consensus meeting will be undertaken to achieve consensus on the COS.

There will be two phases:

1. Identification of an outcome long list through:
  - Systematic literature review to identify clinical and patient-reported outcomes relevant to TBPI care.
  - Semi-structured interviews to identify outcomes important to patients.
  - Reduction of the long list by grouping similar items together into domains and to create questionnaire items for the Delphi.
2. Prioritisation of outcomes through an online three-round Delphi and a consensus meeting to agree the final COS.

### Phase 1: Identification of an outcome long list

The aim of phase 1 is to identify a comprehensive list of outcomes important to patients with TBPI and to clinicians and researchers. This will be achieved through a systematic review of outcomes reported in the literature and interviews with individuals with a TBPI to gain their views on what outcome domains should be measured. Combining the results of the systematic review and patient interviews will generate a long list of outcome domains (eg, pain, movement) to be taken forward to the Delphi study.

#### Systematic review

A systematic review of literature evaluating the effectiveness of surgical and non-surgical interventions for patients with TBPI will be undertaken to identify clinical and patient reported outcomes.

The systematic review will:

1. Identify clinical and patient-reported outcomes reported in studies evaluating treatments in TBPI.
2. Identify measurement tools used to assess outcomes.

With few prospective or randomised trials in this clinical area, the criteria for inclusion will be any controlled and uncontrolled experimental or observational studies evaluating any intervention in adult TBPI including case reports, case series, case studies, prospective and retrospective cohort studies and randomised and non-randomised clinical trials. The definition of an intervention includes any surgical or non-surgical care. Clinical outcomes and outcome measurement tools are those relevant to assessment of patient’s recovery and well-being and will include short-term outcomes including adverse events and complications of surgical and non-surgical care and long-term outcomes, for example, quality of life.

The search will be limited to the last 5 years so that outcomes extracted reflect use in recent studies relating to modern treatment of TBPI. Non-English language publications will be included. Conference abstracts will be excluded due to the high risk of duplication with published studies. Studies will not be excluded because of research quality due to the exploratory nature of the review. For searching, Medical Subject Headings including subheadings, publication types and supplementary concepts will be used. Free text (keywords) will also be applied to the term (see online supplementary appendix A for the MEDLINE(OVID) search strategy).

10.1136/bmjopen-2019-030146.supp1 Supplementary data

Titles, then abstracts will be independently screened by two reviewers. Disagreements will be resolved through discussion with the study management group if necessary. Exclusions, with reasons, will be noted at each stage. Clinical and patient-reported outcomes and patient-reported outcome measures (PROMS) will be extracted from eligible full texts by one reviewer (CM) using a pilot-tested data extraction form. A second reviewer will independently extract outcome data. Dual data extraction will stop when outcomes extracted are identical for the last 10 studies. Study details to be extracted will include authors, date, country, study design, participant demographics (mean age, sex, type of TBPI, participant numbers), reported outcomes, adverse events or complications, outcome definition, outcome measures and time point of measures after injury.

Where a PROM has been used, it will be documented and the original PROM acquired and data extracted as recommended by Macefield *et al*.^37^ The following data will be extracted, verbatim name for PRO scales as termed by PROM developer, verbatim name for single items as termed by PROM developer, all PROM items (scale components and any single items) and details of any additional non-validated questions.

A narrative synthesis will be conducted with results presented in tables. Methodological quality of the included studies will not be assessed as the aim of the review is to identify outcomes reported in intervention studies with TBPI participants regardless of study quality. A summary of all outcomes used will be generated, including frequency analysis and relationship to time and geographic area. A summary of the technique/tools used to assess each outcome will be made.

#### Semi-structured interviews

Semi-structured qualitative interviews will be undertaken to identify and explore outcomes important to patients with a TBPI.

This study will:

- Identify outcomes important to individuals with a TBPI.
- Facilitate understanding of why these outcomes are important.
- Identify appropriate language for outcome domains to be used in the Delphi.
- Facilitate comparison between outcomes identified in the systematic review.

##### Method

The study design was informed by the Consolidated Criteria for Reporting Qualitative Research (COREQ).^38^ The approach will be pragmatic, reflecting the need to identify all outcomes important to individuals with a TBPI.^39^

##### Inclusion criteria

Types of participants

- Adults >18 years of age.
- Patients who have completed or are receiving treatment for TBPI.
- Able to participate in an interview in the English language.

Types of pathology

- TBPI.

Types of intervention

- All surgical and non-surgical interventions for TBPI.

##### Exclusion criteria

Types of participant

- Unable to give informed consent.

Types of pathology

- Other significant comorbidities that could impact cognition or peripheral neurological function, for example, a traumatic brain injury.

##### Sampling

CM will screen the peripheral nerve injury database and peripheral nerve clinic lists at the Queen Elizabeth Hospital Birmingham, a tertiary regional referral centre and specialist unit for patients with TBPI. Potential participants on the peripheral nerve injury database will be sent an invitation letter and asked to contact CM if interested in participating in the study. Potential participants identified on the clinic lists will be approached by a member of the multidisciplinary team (physiotherapist, occupational therapist, surgeon, specialist nurse or senior registrar). The study will be explained, and they will be provided with a patient information leaflet. Patients will have as much time as necessary to consider the study and take the information leaflet home to discuss with family and friends. Patients will be asked to contact CM if they are interested in the study. At this time, the study will be explained again, and any questions will be answered. If the patient is interested in participating, then a convenient time and date for a 1:1 interview will be negotiated. One-to-one interviews will be conducted by a single researcher(CM). A ‘maximum variation sample’ will be sought^40 41^ so that outcomes generated from the interviews cover the spectrum of dysfunction associated with a TBPI. A sampling framework, constructed to reflect important characteristics such as age, sex, type of TBPI, type of interventions received and time since injury, will serve as a guide to recruiting participants for the study. It is envisaged that no more than 15–17 participants will be required for data saturation.^42^

##### Interview location

Interviews will be face to face at a location and time of the patient’s choice which could include the participants home or an interview room at the Queen Elizabeth Hospital Birmingham. Participants will be reimbursed for travel expenses for travelling to and from interview locations.

##### Interview format

The discussion will explore how the injury has impacted on each participant’s life and what their ideal outcomes and expectations are for treatments. A topic guide using open questions and follow-up prompts, developed and piloted with members from the patient advisory group, will be used. This will ensure that the most important aspects are covered while allowing flexibility to explore concepts raised by participants.

##### Data analysis

Data analysis^43 44^ will be undertaken concurrently with data collection through thematic analysis. The interviews will be digitally recorded, and the recordings downloaded to a secure computer then removed from the recording devise. Anonymised recordings will be securely transferred to a transcription company. Anonymised transcripts will be reviewed line by line and words and phrases and passages related to important outcomes following a TBPI or related to treatments will be coded. A second reviewer will code 10% of the transcripts to enhance credibility of the analysis. Differences in codes and emergent themes will be discussed. A member of the patient advisory group will be supported to read a selection of uncoded transcripts and suggest codes or themes. This will assist with the interpretation (from a patient’s perspective) and which may be different to that of the research team. Themes will be discussed and agreed with members of the research team (CJH and JC). The lead author (CM) will carry out interviews until no new themes emerge. From this analysis, we will develop a list of outcomes important to patients with TBPI.

#### Consultation exercise

The aim of this consultation exercise will be to identify, from the list of outcomes identified in the systematic review and the semi-structured interviews, what outcomes should be entered into phase 2 of the study.

##### Study overview and method

The list of outcomes from the systematic review and the interviews with patients will be combined. Each outcome from phase one will be categorised independently by two authors (DP and CM) into one of 36 domains developed by a collaboration between COMET and Cochrane.^45^ Outcome domains like these have previously been used by other COS developers.^46 47^ Outcome domains will include physiological/clinical, function, global quality of life and adverse events. The scope of the outcomes will be defined, and a common language and description will be identified for each of the outcomes through an iterative process between the patient advisors, lead investigator and key members of the research team.

CM will present all outcomes within the COMET and Cochrane framework^45^ at a consultation meeting with the patient advisor, research team and two clinicians. The objective is to ensure clear and appropriate meanings with no duplication. The outcome domains will be reviewed to assess the suitability of the domain name and grouping of outcomes and descriptors. The ‘long list’ of outcomes will be developed into a plain English questionnaire in collaboration with the patient advisory group and the research group for use in the Delphi Study.

### Phase 2: Delphi and consensus meeting

A Delphi method is frequently used to achieve consensus for COS.^48 49^ Time, financial constraints and carbon footprint make this the best option for international expert and patient involvement. A Delphi allows anonymity and gives equal weight to all who participate reducing the potential for individuals or group of individuals being overly influential in the process.^50^

#### Method

We will recruit three panels for the online Delphi.

##### Patient experience panel

Individuals with TBPI will be recruited through the Traumatic Brachial Plexus UK charity website and their social media outputs (Twitter and Facebook). A study video will be hosted on the website and promoted through social media. Potential participants will be asked to contact the chief investigator (CM) if interested in participating. This may favour individuals who have had a TBPI for many years and those accessing online self-help groups, therefore, the peripheral nerve database (n=208) at the Queen Elizabeth Hospital Birmingham will also be screened. A purposive sample from the database will receive an invitation to participate.

##### Surgical panel

Direct approaches to known experts will be made for participation. Researchers will contact the British Association of Orthopaedic Surgeons, British Association of Plastic, Reconstructive and Anaesthetic surgeons and American Society for Surgery of the Hand to act as gatekeepers, seeking to identify experts for the Delphi study. Invitations to participate will be distributed via their websites/newsletter or email. Interested members can then therefore register though this link and confirm their eligibility.

##### Non-surgical panel

Similar to surgical panel experts known to researchers will be approached directly. Working with The British Association of Hand Therapy, Association of Trauma and Orthopaedic Physiotherapists and international equivalents, the advert for the Delphi will be distributed through their websites, mailing lists and newsletters.

Participants (patients and healthcare practitioners) will rate the importance of each outcome through a three-round online Delphi. Participants will receive an email link to the questionnaire embedded in the UK traumatic brachial plexus support website. The Delphi Manager software developed by the COMET Initiative to manage COS development (http://www.comet-initiative.org/delphimanager/) will be used. A paper copy may also be sent if requested. Within the questionnaire, outcomes will be grouped into domains so that similar or related outcomes can be viewed together. Questions, with outcomes written in lay terms, will have medical terms explained on hovering with the mouse. Participants will rate the importance of each outcome on a nine-point Likert scale as recommended by the Grading of Recommendations Assessment, Development and Evaluation working group,^51^ 1=not important and 9=critical for each outcome. Participants will have the option to add additional outcomes in round 1. These will be added to the second-round questionnaire if deemed ‘new’ and within the scope of the COS. After each round, results will be fed back to the participants within subsequent questionnaires. Participants will be able to review:

- The score they gave the outcome in the previous round.
- The mean score given to that outcome by each stakeholder group including their own.
- Anonymised free-text comments for any outcome.

This will allow participants to reflect, review and change their scores from the previous rounds. The method of feedback is important so will be discussed, piloted and refined with the patient and clinical advisory group.

All outcomes from round 1 will remain in round 2 to allow participants to reconsider in the light of feedback attached. Items from round 2 will continue to the next round if any item scores between 7 and 9 (important) by 50% or over and between 1 and 3 (not important) by less than 15% in any stakeholder group. Outcomes, not meeting the criteria, will be discarded. Participants will then rescore each outcome retained in round 3.

All outcomes which meet the ‘Consensus IN’ criteria (see box 1) in any stakeholder group will be taken forward for ratification at the consensus meeting. All outcomes which meet the criteria ‘Consensus OUT’ in all stakeholder groups will not be taken forward to the consensus meeting. Where ‘NO Consensus’ has occurred, the results of these outcomes in each stakeholder group will be taken forward to the consensus meeting and presented for discussion.

Box 1 **Consensus criteria**

Consensus IN (consensus that outcome should be included in the core of outcome set (COS))—70% or more participants scoring an outcome as critically important (7–9) and <15% or fewer participants rate the outcome as limited importance (1–3).

Consensus OUT (consensus that outcome should not be included in the COS)—70% or more participants scoring an outcome as limited importance (1–3) and <15% of participants scoring an outcome as very important (7–9).

NO consensus (uncertainty about importance of outcome)—anything else.

#### Sample size

There is no consensus regarding the optimal sample size for a Delphi study; therefore, recruitment will be based on previous Delphi studies.^52 53^ Each panel will include a minimum of 15–20 participants. The requirement to complete all rounds of the study will be emphasised to limit attrition while adhering to ethical principles.^52^

#### Consensus meeting

##### Overview

A consensus meeting will be held with patients and healthcare professionals to finalise the COS. We will aim to gather approximately 20–25 participants with an equal representation from each stakeholder group. The objective of the consensus meeting is to discuss outcomes for which there was disagreement in round 3 of the Delphi study and to validate and agree on a final list of outcomes^35^ which will constitute the COS.

The results from each round of the Delphi study will be presented and the consensus results from round 3 of the Delphi. It will then be proposed that any outcome categorised as ‘consensus IN’ across all stakeholder groups be included in the final COS, and any outcome categorised as ‘consensus OUT’ across all stakeholder groups be excluded. Attendees will vote anonymously on electronic keypads to accept this proposal or to suggest outcomes from this group which necessitate further discussion.

All other outcomes including those categorised as ‘consensus IN’ or ‘consensus OUT’ by one or two stakeholder groups and those categorised as ‘NO consensus’ will then be discussed, and further rounds of voting will be used to agree the final COS. The purpose of the meeting is to ratify the final outcome set; therefore, the agenda of the meeting and processes used will be in part dependent on the consensus achieved through the Delphi study.

##### Consensus rules and voting

Consensus will be defined as being reached when less than 30% of voters disagree (ie, 70% agree or are unsure). Voting will be anonymous using electronic handsets and voting software, with real-time results fed back to the group once voting is closed. All meeting participants will be permitted to vote (observers, study management group and facilitators will be excluded from voting).

##### Recruitment

All participants who have completed the online Delphi will be able to register their interest in participation in the consensus meeting (tick box on Delphi registration page). A purposive sample of participants from all stakeholder groups (patients, surgical and non-surgical healthcare professionals), who have registered their interest and completed all rounds of the Delphi, will be invited to attend the consensus meeting.

#### Patient and public involvement

Patient and public have been involved in the inception and design of the study. We received input from patients with TBPI on the design of all patient facing documents. A user-led organisation (Trauma Brachial Plexus Injury Group UK) has acted as a collaborator. We have carefully assessed the burden of the project on study participants. We intend to disseminate the main results to study participants and other patients with TBPI and will seek patient and public involvement in the development of an appropriate method of dissemination.

## Ethics and dissemination

All interview participants will provide informed written consent. Consent will be taken as implicit for Delphi participants, registering via the website and submitting completed questionnaires.

The protocol, we propose, observes COMET and COS-STAD recommendations^35 36^ and represents a robust method for COS development. There will be active participation of patients, international healthcare professionals and academics in each phase. In addition, a patient and a clinical advisory group will oversee the project. This will ensure that outcomes important to all stakeholders are represented in the final COS.

Once the final COS is agreed, further work is planned to develop a core outcome measurement instrument set. This future work will aim to recommend a measurement instrument for each outcome in the COS. The systematic review in this protocol will have identified existing measurement instruments. Guidance from COS for the selection of health Measurement INstruments^54^ and Prinsen *et al* 55 will be then followed to identify the measurement instruments with the highest validity, reliability and responsiveness in the TBPI population.

Engaging a wide range of international participants in all stakeholder groups will facilitate dissemination and uptake of the COS in this area of healthcare in the future. The COS development has been registered on COMET, an international public database. Successful implementation of COS in other healthcare areas has resulted in a significant change in the quality and relevance of research and enhanced clinical practice globally.^56^ It is anticipated that a COS for TBPIs will do the same and improve clinical decision making, patient care and outcomes in this area.

## Supplementary Material

Reviewer comments
