# Supplementary material for: Development of a core outcome set for traumatic brachial plexus injuries (COMBINE): study protocol
Source: BMJ Open. 2019 Jun 14;9(6):e030146. doi: 10.1136/bmjopen-2019-030146 (PMC6575635; doi:10.1136/bmjopen-2019-030146)
Supplement: Supplementary data [file bmjopen-2019-030146supp001.pdf]

Supplementary File 1

Search strategy COMBINE systematic review

MEDLINE (OVID)

1.(brachial plexus adj3 injur*).mp. [mp=title, abstract, original title, name of substance word, subject heading word, floating sub-heading word, keyword heading word, protocol supplementary concept word, rare disease supplementary concept word, unique identifier, synonyms]

2 (brachial plexus adj3 pals*).mp. [mp=title, abstract, original title, name of substance word, subject heading word, floating sub-heading word, keyword heading word, protocol supplementary concept word, rare disease supplementary concept word, unique identifier, synonyms]

3 (brachial plexus adj3 lesion*).mp. [mp=title, abstract, original title, name of substance word, subject heading word, floating sub-heading word, keyword heading word, protocol supplementary concept word, rare disease supplementary concept word, unique identifier, synonyms]

4 brachial plexopath*.mp. [mp=title, abstract, original title, name of substance word, subject heading word, floating sub-heading word, keyword heading word, protocol supplementary concept word, rare disease supplementary concept word, unique identifier, synonyms]

5 (brachial plexus adj3 traction*).mp. [mp=title, abstract, original title, name of substance word, subject heading word, floating sub-heading word, keyword heading word, protocol supplementary concept word, rare disease supplementary concept word, unique identifier, synonyms]

6 (brachial plexus adj3 avulsion*).mp. [mp=title, abstract, original title, name of substance word, subject heading word, floating sub-heading word, keyword heading word, protocol supplementary concept word, rare disease supplementary concept word, unique identifier, synonyms]

7 Brachial Plexus/in, pd, su, tr [Injuries, Pharmacology, Surgery, Transplantation]

8 1 or 2 or 3 or 4 or 5 or 6 or 7

9 limit 8 to (humans and "all adult (19 plus years)")

10. limit 9 to yr="2013 -Current"
