# Supplementary material for: Development of a core outcome set for traumatic brachial plexus injuries (COMBINE): study protocol
Source: BMJ Open. 2019 Jun 14;9(6):e030146. doi: 10.1136/bmjopen-2019-030146 (PMC6575635; doi:10.1136/bmjopen-2019-030146)
Supplement: Reviewer comments [file bmjopen-2019-030146.reviewer_comments.pdf]

PEER REVIEW HISTORY

BMJ Open publishes all reviews undertaken for accepted manuscripts. Reviewers are asked to complete a checklist review form (**http://bmjopen.bmj.com/site/about/resources/checklist.pdf**) and are provided with free text boxes to elaborate on their assessment. These free text comments are reproduced below.

ARTICLE DETAILS

**TITLE (PROVISIONAL)**	Development of a core outcome set for traumatic brachial plexus injuries (COMBINE): a study protocol
**AUTHORS**	Miller, Caroline; cross, jane; Power, Dominic; Kyte, Derek; Jerosch-Herold, Christina

This article was not externally reviewed at BMJ Open. Protocols that have been independently assessed prior to submission to BMJ Open are usually fast-tracked to publication on the grounds that further substantial changes will not be possible. This independent assessment will usually be external, independent review for both a substantial grant award from a non-commercial or government funder and ethics approval.
